# An event generator for same-sign W-boson scattering at the LHC including electroweak corrections

**DOI:** 10.1140/epjc/s10052-019-7290-6

**Published:** 2019-09-24

**Authors:** Mauro Chiesa, Ansgar Denner, Jean-Nicolas Lang, Mathieu Pellen

**Affiliations:** 1 Institut für Theoretische Physik und Astrophysik, Universität Würzburg, Emil-Hilb-Weg 22, 97074 Würzburg, Germany; 2 Universität Zürich, Physik-Institut, 8057 Zürich, Switzerland; 3 University of Cambridge, Cavendish Laboratory, Cambridge, CB3 0HE UK

## Abstract

In this article we present an event generator based on the Monte Carlo program Powheg in combination with the matrix-element generator Recola. We apply it to compute NLO electroweak corrections to same-sign W-boson scattering, which have been shown to be large at the LHC. The event generator allows for the generation of unweighted events including the effect of the NLO electroweak corrections matched to a QED parton shower and interfaced to a QCD parton shower. In view of the expected experimental precision of future measurements, the use of such a tool will be indispensable.

## Introduction

One way of probing the mechanism of electroweak (EW) symmetry breaking and the properties of the Higgs boson is through the detailed study of the scattering of EW vector bosons at colliders. Among the vector-boson scattering (VBS) processes, the same-sign leptonic signature is probably the golden channel at the Large Hadron Collider (LHC). Having the best signal over background ratio, due to its very small Standard Model (SM) background and its relatively large cross section, it has been the first of the VBS processes measured at the LHC [1–5] and in the coming years its measurement is expected to be accurate at a few-per-cent level [6]. At this level of accuracy, higher-order corrections are mandatory for theoretical predictions. For this particular process, the next-to-leading-order (NLO) EW corrections have been found to be particularly large [7] and even the largest NLO contributions [8] for the full process $\text {p} \text {p} \rightarrow \mu ^+ \nu _{\mu } \text {e} ^+ \nu _{\text {e}}\text {j} \text {j}$. This renders the availability of these corrections in appropriate tools one of the priority tasks in the quest for the precise measurements of this process.

The scattering of same-sign W bosons is the VBS process that draws most theoretical interest. While it is the simplest VBS channel to compute in terms of the number of Feynman diagrams and partonic channels, it features many characteristics of other VBS channels. Several years ago, the QCD corrections in the VBS approximation [9, 10] have been obtained [11, 12] and implemented in the parton-level Monte Carlo program VBFNLO [13–15]. These calculations have subsequently been matched to QCD parton shower (PS) [16] using the program Powheg-Box-V2 [17–19]. These approximate computations have been recently compared against the full computation [8], and the agreement turned out to be satisfactory given the current experimental precision [20]. The computation of the full NLO corrections to the process $\text {p} \text {p} \rightarrow \text {e} ^+ \nu _{\text {e}} \mu ^+ \nu _{\mu } \text {j} \text {j}$ [8] revealed that the EW corrections are the dominant NLO contributions for this channel. Indeed, as argued in Ref. [7] and confirmed in the WZ channel [21], large EW corrections are an intrinsic feature of VBS at the LHC.

In this article, we introduce a new generator based on the Monte Carlo program Powheg [17–19] in combination with the matrix-element generator Recola [22, 23]. The capabilities of Powheg+Recola are exemplified by the computation of NLO EW corrections matched to QED PS and supplemented by QCD PS for the processes $\text {p} \text {p} \rightarrow \ell ^\pm _1 \nu _{\ell _1} \ell ^\pm _2 \nu _{\ell _2}\text {j} \text {j}$ defined at $\mathcal {O}\left( \alpha ^6\right)$, where $\ell _1, \ell _2 = \text {e}, \mu$. To date, this computation is one of the most complicated NLO EW calculations performed with a public tool along with the recent off-shell tri-boson computation of Ref. [24]. It is a $2\rightarrow 6$ process involving six external charged particles and up to four resonances. In that respect the use of the newly developed Powheg-Box-Res [25] which accounts for resonant histories is particularly valuable. This development of Powheg has already been applied to the calculation of the NLO QCD corrections matched to QCD PS to single-top [25] and top-pair production [26], and to the calculation of the NLO QCD+EW corrections matched to both QCD and QED PS for processes like Drell–Yan [27],^1^ and HV+jet ($V=$W, Z) production [29].

The Powheg+Recola generator computes NLO EW corrections at order $\mathcal {O}\left( \alpha ^7\right)$ for all possible lepton-flavour combinations of the same-sign W-boson scattering channel at the LHC. In addition to fixed-order predictions, one can generate unweighted events including the effect of the NLO EW corrections that can be passed to QED and QCD shower Monte Carlo programs in order to reach the NLO EW matched to QED PS accuracy. In particular, we provide an interface to the program PYTHIA [30, 31]. The code can be found under the WWW address:

http://powhegbox.mib.infn.it/.

In addition to presenting the code, we provide some phenomenological results. In particular, we present for the first time the NLO EW corrections to the $\text {p} \text {p} \rightarrow \text {e} ^- {\bar{\nu }}_{\text {e}} \mu ^- {\bar{\nu }}_{\mu } \text {j} \text {j}$ signature. As expected, while the total rate is very different from the one for the $\text {p} \text {p} \rightarrow \text {e} ^+ \nu _{\text {e}} \mu ^+ \nu _{\mu } \text {j} \text {j}$ signature, the relative EW corrections are very similar and differ only marginally. We also show illustrative predictions at NLO EW+PS accuracy.

This article is organised as follows: in Sect. 2, the process to be studied is defined. Section 3 is devoted to the description of the implementation. The set-up used for the prediction is described in Sect. 4. Finally, Sect. 5 contains results as well as recommendations for the use of the present tool. The article ends with a summary and concluding remarks in Sect. 6.

## Description of the process

The computation of the EW corrections to same-sign W-boson scattering closely follows the computation of the specific channel $\text {p} \text {p} \rightarrow \text {e} ^+ \nu _{\text {e}} \mu ^+ \nu _{\mu } \text {j} \text {j}$ published in Refs. [7, 8]. The code presented here allows to compute all combinations of lepton flavours for the same-sign WW channel, *i.e.* the four independent hadronic processes:

1 $$\begin{aligned}&\text {p} \text {p} \rightarrow \text {e} ^+ \nu _{\text {e}} \mu ^+ \nu _{\mu } \text {j} \text {j}, \end{aligned}$$

2 $$\begin{aligned}&\text {p} \text {p} \rightarrow \text {e} ^- \nu _{\text {e}} \mu ^- \nu _{\mu } \text {j} \text {j}, \end{aligned}$$

3 $$\begin{aligned}&\text {p} \text {p} \rightarrow \text {e} ^+ \nu _{\text {e}} \text {e} ^+ \nu _{\text {e}} \text {j} \text {j}, \end{aligned}$$

4 $$\begin{aligned}&\text {p} \text {p} \rightarrow \text {e} ^- \nu _{\text {e}} \text {e} ^- \nu _{\text {e}} \text {j} \text {j} . \end{aligned}$$

As both muons and electrons are considered massless, the processes $\text {p} \text {p} \rightarrow \mu ^+ \nu _{\mu } \mu ^+ \nu _{\mu } \text {j} \text {j}$ and $\text {p} \text {p} \rightarrow \mu ^- \nu _{\mu } \mu ^- \nu _{\mu } \text {j} \text {j}$ can directly be obtained from processes (3) and (4), respectively.

In the leading-order (LO) process we take into account all contributions at order $\mathcal {O}\left( \alpha ^6 \right)$. This gauge-invariant quantity includes besides the VBS contribution all contributions with less than two resonant W bosons and contributions to triple W-boson production. Nevertheless, in the rest of this article, we often refer to the full EW contribution of order $\mathcal {O}\left( \alpha ^6\right)$ as VBS. The NLO EW corrections are defined to incorporate all contributions of order $\mathcal {O}\left( \alpha ^7 \right)$ and are made of real radiation and virtual contributions, the sum of both being infrared finite. Photon-induced contributions are not included in the present computation, as they have been shown to be at the per-cent level [8]. In the real corrections, only photon radiation is taken into account, while heavy gauge-boson radiation is not incorporated. This effect is of the order of few per cent in the phase-space region defined by the typical VBS event-selection cuts at the LHC [32].

## Details of the calculation

### Powheg

The POWHEG algorithm was developed in Refs. [17, 18] for the generation of events at NLO QCD accuracy matched to QCD PS in order to avoid the double counting of the $\mathcal {O}\left( \alpha _\text {s} \right)$ contributions coming from PS. It is based on the Frixione-Kunszt-Signer (FKS) subtraction method [33, 34] for the separation of the real radiation processes into singular regions (*i.e.* the regions of phase space where one parton in the considered real process becomes soft and/or collinear to another parton) and for the integration of the real corrections. Events are generated according to the formula [18, 19]:

5 $$\begin{aligned} {\mathrm {d}}\sigma= & {} \sum _{f_{\mathrm {b}}} \bar{B}^{f_{\mathrm {b}}}(\mathbf {\Phi }_n) \, {\mathrm {d}}\mathbf {\Phi }_n \left\{ \Delta ^{f_{\mathrm {b}}} (\mathbf {\Phi }_n,p_{\mathrm {T}}^{\mathrm {min}}) \right. \nonumber \\&\left. + \sum _{\alpha _r \in \{ \alpha _r | f_{\mathrm {b}}\} } \frac{ \left[ {\mathrm {d}}\Phi _{\mathrm {rad}} \, \theta (k_{\mathrm {T}}- p_{\mathrm {T}}^{\mathrm {min}}) \, \Delta ^{f_{\mathrm {b}}}(\mathbf {\Phi }_n, k_{\mathrm {T}}) \, R(\mathbf {\Phi }_{n+1}) \right] _{\alpha _r}^{\bar{\mathbf {\Phi }}_n^{\alpha _r} = \mathbf {\Phi }_n} }{ B^{f_{\mathrm {b}}} (\mathbf {\Phi }_n)} \right\} \, . \nonumber \\ \end{aligned}$$

In Eq. (5) the index $f_{\mathrm {b}}$ runs over the possible underlying Born (UB) processes under consideration, $\bar{B}^{f_{\mathrm {b}}}$ is the corresponding effective squared matrix element including all NLO contributions, $B^{f_{\mathrm {b}}}$ and *R* are the Born and real radiation squared matrix elements with the corresponding *n* and $n+1$-body kinematics $\mathbf {\Phi }_{n}$ and $\mathbf {\Phi }_{n+1}$, and ${\mathrm {d}}\Phi _{\mathrm {rad}}$ is the phase-space volume element for the emitted parton in the real radiation processes. The term in curly brackets represents the probability of emitting one parton with transverse momentum $k_{\mathrm {T}}$ with respect to the corresponding emitter parton from each of the singular regions $\alpha _r$ that are mapped on the considered UB process $f_{\mathrm {b}}$, and $\bar{\mathbf {\Phi }}_n^{\alpha _r}$ denotes the phase-space parametrisation corresponding to the mapping in the singular region $\alpha _r$. For each UB process $f_{\mathrm {b}}$, the POWHEG Sudakov form factor is the product of individual form factors corresponding to the singular regions projecting on the UB $f_{\mathrm {b}}$ and in the notation of Refs. [18, 19] reads

6 $$\begin{aligned} \Delta ^{f_{\mathrm {b}}}(\mathbf {\Phi }_n, k_{\mathrm {T}})=\prod _{\alpha _r\, \in \{ \alpha _r | f_{\mathrm {b}}\}} \Delta ^{f_{\mathrm {b}}}_{\alpha _r} (\mathbf {\Phi }_n, k_{\mathrm {T}}), \end{aligned}$$

where

7 $$\begin{aligned}&\Delta ^{f_{\mathrm {b}}}_{\alpha _r} (\mathbf {\Phi }_n, k_{\mathrm {T}})\nonumber \\&\quad = \exp \Biggl \{-\int \frac{ \left[ {\mathrm {d}}\Phi _{\mathrm {rad}} \, \theta \left( p_{\mathrm {T}}(\mathbf {\Phi }_{n+1})-k_{\mathrm {T}}\right) \, R(\mathbf {\Phi }_{n+1}) \right] _{\alpha _r}^{\bar{\mathbf {\Phi }}_n^{\alpha _r} = \mathbf {\Phi }_n} }{ B^{f_{\mathrm {b}}} (\mathbf {\Phi }_n)} \Biggr \}. \nonumber \\ \end{aligned}$$

The Sudakov form factors $\Delta ^{f_{\mathrm {b}}}_{\alpha _r}$ in Eqs. (6) and (7) are used to generate one radiation from each of the singular regions: the hardest radiation is then written in the Les Houches Event (LHE) and the corresponding $k_{\mathrm {T}}$ is set as the starting scale for the PS (which should be either ordered in $k_{\mathrm {T}}$ or vetoed in the phase-space region harder than the POWHEG radiation). The algorithm is implemented in the Powheg-Box-V2 code [17–19]: this framework allows the users to implement their own Monte Carlo generators for specific processes upon providing the list of the Born and real processes together with the corresponding Born, virtual, and real matrix elements.

In Ref. [25] a new version of the POWHEG algorithm specifically designed for the treatment of processes involving unstable particles was developed and implemented in the Powheg-Box-Res code. On the one hand, it uses a modified version of the FKS subtraction method to improve the integration of the NLO normalization in the presence of resonances and, on the other hand, it allows to generate events with more than one radiation. Instead of looking for the global hardest radiation, the code loops over all possible resonances of the UB under consideration (plus the rest of the hard production process besides the resonances as an additional “resonance”) and for each resonance the hardest among the radiations generated by this resonance is written in the LHE and the corresponding $k_{\mathrm {T}}$ is set as the starting scale for the PS evolution of the particles belonging to the selected resonance. The mappings in Powheg-Box-Res are constructed in such a way that the invariant masses of the resonances are preserved. We used this framework for the implementation of the process $\text {p} \text {p} \rightarrow \ell ^\pm _1 \nu _{\ell _1} \ell ^\pm _2 \nu _{\ell _2}\text {j} \text {j}$ with $\ell _1, \ell _2 = \text {e}, \mu$.

The POWHEG algorithm was extended to the calculation of NLO QCD$+$NLO EW corrections matched to both QCD and QED PS in Refs. [35, 36]. However, this generalization only works for processes where the possible UB processes are univocally defined by their flavour structure, *i.e.* there are no UB processes sharing the same flavour structure but with different order in the coupling constants, which is not the case for VBS, where the $\mathcal {O}\left( \alpha _\text {s} \right)$ corrections to the $\mathcal {O}\left( \alpha ^6 \right)$ Born cannot be disentangled from the $\mathcal {O}\left( \alpha \right)$ corrections to the $\mathcal {O}\left( \alpha _\text {s} \alpha ^5 \right)$ one. For this reason we consider only the $\mathcal {O}\left( \alpha ^6 \right)$ Born processes and compute only the NLO EW corrections matched to QED PS, leaving the general implementation of the NLO QCD+NLO EW corrections to a future work. This choice is justified by the relative importance of the $\mathcal {O}\left( \alpha ^6 \right)$ Born processes and by the size of the NLO EW corrections compared the NLO QCD ones. In Sect. 5.4 we provide a recipe to combine our predictions with the ones at NLO QCD accuracy matched to PS that already exist in the literature.

### Recola

The matrix elements required in Powheg-Box are obtained from Recola  [22, 23], a high-performance one-loop matrix-element generator for the Standard Model. Recola generates all the needed ingredients for one-loop computations, such as (un-)polarised or colour(-spin)-correlated tree-and one-loop amplitudes for arbitrary processes. The processes are generated on request and on-the-fly in memory, *i.e.* without generating process source code. The evaluation is purely numerical and recursive using Berends–Giele-like recursion at LO [37]. At NLO, it uses the algorithm for tensor coefficients by A. van Hameren [38] suitably extended for the complete SM [22]. Tensor integrals are obtained by means of the tensor-integral library Collier  [39]. Recola supports standard schemes for the renormalisation of the strong and EW couplings. Physical fields are renormalized in the complete on-shell scheme with unstable particles treated according to the complex-mass scheme [40–42]. Recola has passed several non-trivial tests, and we simply mention the technical comparison which has been performed in Ref. [43] for di-boson production at NLO EW accuracy.

In the interface to Powheg-Box, the new version Recola2 [44, 45] is used, which is fully backwards compatible, but allows for models beyond the SM. Among the improvements in this new version, there is a significant reduction of the memory consumption of processes hold in memory. This has been achieved, on the one hand, by optimising the memory management in Recola2 for single processes and, on the other hand, by linking processes related via crossing symmetry. From the user point of view all processes are defined as before, and Recola2 takes care of all necessary crossings of the kinematics automatically. For instance, in the case of VBS with the $\text {e} ^+ \nu _\text {e} \mu ^+ \nu _\mu$ leptonic final state considered in this article, Recola2 internally generates only two types of amplitudes,

8 $$\begin{aligned}&0 \rightarrow \text {e} ^+ \nu _\text {e} \mu ^+ \nu _\mu {\bar{\text {c}}} {\bar{\text {u}}} \text {s} \text {d}, \nonumber \\&0 \rightarrow \text {e} ^+ \nu _\text {e} \mu ^+ \nu _\mu {\bar{\text {u}}} {\bar{\text {u}}} \text {d} \text {d}, \end{aligned}$$

but calculates all the distinct channels (as defined by the user). In addition to the improvements of Recola2 , the cache management of Collier has been refined. From version 1.2.3 onwards, the memory consumption has been considerably reduced for complicated processes.

Like Recola, Recola2 has passed non-trivial checks and has been cross-checked against various independent calculations. As an additional feature, Recola2 can perform computation in the Background-Field Method [46–50] which allows for powerful checks of virtual amplitudes when comparing to the usual computation method in the ’t Hooft–Feynman gauge.

### Powheg+Recola

For each of the hadronic processes $\text {p} \text {p} \rightarrow \ell ^\pm _1 \nu _{\ell _1} \ell ^\pm _2 \nu _{\ell _2}\text {j} \text {j}$ with $\ell _1, \ell _2 = \text {e}, \mu$, there are 12 partonic processes (see Table 1 of Ref. [8]). Several of them share the same matrix element. Upon applying the relevant parton-distribution function (PDF) factor, these can be merged. Using crossing of particles in the initial state, one can reduce the set of matrix elements to be declared in POWHEG to seven. For the two sets of differently charged final-state leptons, these are given by:

9 $$\begin{aligned} \begin{array}{ll} {\bar{\text {d}}} {\bar{\text {d}}} \rightarrow \ell ^+_1 \nu _{\ell _1} \ell ^+_2 \nu _{\ell _2} {\bar{\text {u}}} {\bar{\text {u}}}, &{}\quad {\bar{\text {u}}} {\bar{\text {u}}} \rightarrow \ell ^-_1 \nu _{\ell _1} \ell ^-_2 \nu _{\ell _2} {\bar{\text {d}}} {\bar{\text {d}}}, \\ {\bar{\text {d}}} \text {u} \rightarrow \ell ^+_1 \nu _{\ell _1} \ell ^+_2 \nu _{\ell _2} {\bar{\text {u}}} \text {d}, &{}\quad {\bar{\text {u}}} \text {d} \rightarrow \ell ^-_1 \nu _{\ell _1} \ell ^-_2 \nu _{\ell _2} {\bar{\text {d}}} \text {u}, \\ \text {u} \text {u} \rightarrow \ell ^+_1 \nu _{\ell _1} \ell ^+_2 \nu _{\ell _2} \text {d} \text {d}, &{}\quad \text {d} \text {d} \rightarrow \ell ^-_1 \nu _{\ell _1} \ell ^-_2 \nu _{\ell _2} \text {u} \text {u}, \\ {\bar{\text {s}}} {\bar{\text {d}}} \rightarrow \ell ^+_1 \nu _{\ell _1} \ell ^+_2 \nu _{\ell _2} {\bar{\text {c}}} {\bar{\text {u}}} , &{}\quad {\bar{\text {c}}} {\bar{\text {u}}} \rightarrow \ell ^-_1 \nu _{\ell _1} \ell ^-_2 \nu _{\ell _2} {\bar{\text {s}}} {\bar{\text {d}}} , \\ {\bar{\text {s}}} \text {u} \rightarrow \ell ^+_1 \nu _{\ell _1} \ell ^+_2 \nu _{\ell _2} {\bar{\text {c}}} \text {d}, &{}\quad {\bar{\text {c}}} \text {d} \rightarrow \ell ^-_1 \nu _{\ell _1} \ell ^-_2 \nu _{\ell _2} {\bar{\text {s}}} \text {u}, \\ \text {u} \text {c} \rightarrow \ell ^+_1 \nu _{\ell _1} \ell ^+_2 \nu _{\ell _2} \text {d} \text {s}, &{}\quad \text {d} \text {s} \rightarrow \ell ^-_1 \nu _{\ell _1} \ell ^-_2 \nu _{\ell _2} \text {u} \text {c}, \\ \text {u} {\bar{\text {d}}} \rightarrow \ell ^+_1 \nu _{\ell _1} \ell ^+_2 \nu _{\ell _2} {\bar{\text {c}}} \text {s}, &{}\quad {\bar{\text {c}}} \text {s} \rightarrow \ell ^-_1 \nu _{\ell _1} \ell ^-_2 \nu _{\ell _2} \text {u} {\bar{\text {d}}} . \end{array} \end{aligned}$$

Among these, the first three and the last four partonic processes are related by initial–final-state crossing. Therefore, even if declared in the interface, only the amplitudes of Eq. (8) have to be generated by Recola2.

**Fig. 1:**
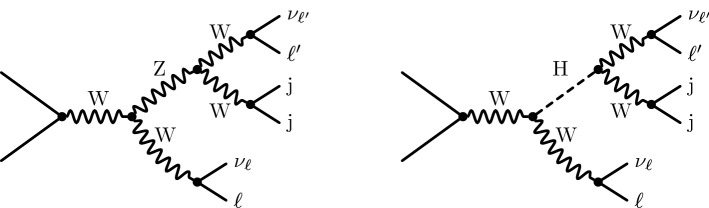
Graphical representation of the two diagrams with the highest number of massive resonances for $\text {p} \text {p} \rightarrow \ell ^\pm _1 \nu _{\ell _1} \ell ^\pm _2 \nu _{\ell _2}\text {j} \text {j}$. The resonances of any other contribution can be matched to one of the resonances in these two diagrams

The partonic processes described in Eq. (9), can be divided into three categories according to their resonance structure. Some processes involve only *t*-channel (and *u*-channel) diagrams, some involve only *s*-channel diagrams, and some receive contributions from *s*- and *t*-channel diagrams (see Table 1 in Ref. [8]). The *t*-channel diagrams have a simple resonance structure with only two resonant W bosons which decay leptonically. For *s*-channel diagrams, the resonance structure can be more intricate. The two most complicated resonance structures for the given hadronic processes are displayed in Fig. 1, and each one contains five potentially resonant massive propagators in total. One of them can either be a Z boson or a Higgs boson. Any other occurring resonance structure can be obtained from one of the resonance structures in Fig. 1 by discarding one or several resonant propagators.

As mentioned in Sect. 2, our generator can compute the four hadronic processes (1)–(4) covering all possible same-sign W-scattering channels. In addition, we provide an interface to PYTHIA [30, 31] to perform the QED as well as the QCD PS matching. Besides the PS evolution, PYTHIA provides hadronisation and decays of unstable hadrons. The Powheg-Box-Res matching strategy described in Sect. 3.1 is used for the final-state QED PS from the resonance decay products. However, for the QCD and QED PS evolution of the coloured partons we chose as starting scale the geometric average of the transverse momenta of the partonic jets in the LHE. This choice is motivated by the fact that the NLO QCD corrections to VBS are not included in our calculation and thus there is no dynamical competition between QED and QCD radiation at the event generation level. As a consequence, setting the starting scale for both the QED and the QCD PS to the $k_{\mathrm {T}}$ of the photon generated by POWHEG from the coloured partons will unphysically suppress the phase space for the QCD PS radiation. The scale for the QCD PS is set to $\sqrt{ p_{\text {T},\text {j} _1} p_{\text {T},\text {j} _2} }$ rather than to the partonic centre of mass of the event since the former definition is directly related to the relevant kinematical invariants for the QCD corrections, while the latter choice would lead to an overestimate of the QCD PS contributions as pointed out in Ref. [51] for Higgs production in vector-boson fusion. This means that the maximum virtuality for the QCD and QED radiation is $\sqrt{ p_{\text {T},\text {j} _1} p_{\text {T},\text {j} _2} }$, but the QED radiation is vetoed if $p_{\text {T},\gamma } > p_{\text {T},\mathrm Powheg}$ in order to avoid double-counting. For this publication we have used Pythia version 8.235.

The code is available at:

http://powhegbox.mib.infn.it/.

More details about the actual settings and instructions how to run the code are given in the user manual available within the package. Finally, despite the interface between Powheg and Recola not being fully general, it can serve as a template for the computation of many other processes at NLO EW accuracy matched to QED PS. As mentioned in Sect. 3.1, the simultaneous inclusion of NLO QCD and NLO EW corrections requires several modifications in the non-process-specific part of the Powheg-Box code and is left for future work. Note that there already exists a fully general interface between Recola and the Monte Carlo generator Sherpa [52–54]. It is dubbed Sherpa+Recola [55] and allows to compute NLO QCD+EW corrections at fixed order for arbitrary processes.

## Input parameters and selection cuts

All input parameters have been chosen as in Refs. [7, 8]. While these are not the most up-to-date parameters, they allow a simple comparison against the existing computation (these parameters can be changed at will in the code). For completeness we reproduce them here.

The centre-of-mass energy of the simulated hadronic scattering processes is $\sqrt{s} = 13 \,\text {TeV}$ at the LHC. We use the NNPDF3.0QED PDF set [56, 57]^2^ with five massless flavours, NLO QCD evolution, and a strong coupling constant $\alpha _\text {s} \left( M_\text {Z} \right) = 0.118$. For same-sign W-boson scattering, there are no bottom (anti)quarks in the initial or final state, since these would lead to top quarks in the final state that give rise to a different experimental signature. Singularities arising from collinear initial-state radiation are factorised according to the ${\overline{\mathrm{MS}}}$ scheme as done in the NNPDF set.

For the massive particles, the following masses and decay widths are used:

10 $$\begin{aligned} \begin{array}{ll} m_\text {t} = 173.21\,\text {GeV}, &{} \quad \Gamma _{\text {t}} = 0 \,\text {GeV}, \\ M_{\text {Z}}^\text {OS} = 91.1876\,\text {GeV}, &{} \quad \Gamma _\text {Z} ^\text {OS} = 2.4952\,\text {GeV}, \\ M_\text {W} ^\text {OS} = 80.385\,\text {GeV}, &{} \quad \Gamma _{\text {W}}^\text {OS} = 2.085\,\text {GeV}, \\ M_\mathrm{H} = 125.0\,\text {GeV}, &{} \quad \Gamma _{\text {H}} = 4.07 \times 10^{-3}\,\text {GeV}. \end{array} \end{aligned}$$

All fermions are considered as massless particles, with the only exception of the top quark. The conversion into the pole values of the masses and widths for the gauge bosons ($V=\text {W},\text {Z}$) from the measured on-shell (OS) values is obtained according to Ref. [59]:

11 $$\begin{aligned} M_V= & {} M_{V}^\text {OS}/\sqrt{1+(\Gamma _{V}^\text {OS}/M_{V}^\text {OS})^2}\,, \qquad \nonumber \\ \Gamma _V= & {} \Gamma _{V}^\text {OS}/\sqrt{1+(\Gamma _{V}^\text {OS}/M_{V}^\text {OS})^2}. \end{aligned}$$

For the mass and width of the Higgs boson we follow the recommendations of Ref. [60]. The EW coupling is obtained in the $G_\mu$ scheme (see *e. g.* Refs. [61–63]) according to

12 $$\begin{aligned} \alpha = \frac{\sqrt{2}}{\pi } G_{\mu } M_\mathrm{W}^2 \left( 1-\frac{M_\mathrm{W}^2}{M_\mathrm{Z}^2} \right) , \end{aligned}$$

with

13 $$\begin{aligned} G_{\mu } = 1.16637\times 10^{-5}\,\text {GeV} ^{-2}. \end{aligned}$$

The renormalisation and factorisation scales have been set to

14 $$\begin{aligned} \mu _\mathrm{ren} = \mu _\mathrm{fac} = M_\text {W}. \end{aligned}$$

We consider an event selection that mimics the experimental one of Refs. [1, 2]. The fiducial region is defined by the presence of two prompt charged leptons ($\ell =\text {e},\mu$) with same charge, missing momentum and at least two QCD jets passing the following cuts:

15 $$\begin{aligned}&p_{\text {T},\ell }> 20\,\text {GeV}, \quad |y_{\ell }| < 2.5, \quad \Delta R_{\ell \ell }> 0.3, \nonumber \\&p_\mathrm{T, miss} > 40\,\text {GeV}, \end{aligned}$$

16 $$\begin{aligned}&p_{\text {T},\text {j}}> 30\,\text {GeV}, \quad |y_\text {j} | < 4.5, \quad \Delta R_{\text {j} \ell } > 0.3, \end{aligned}$$

17 $$\begin{aligned}&m_{\text {j} \text {j}}> 500\,\text {GeV}, \quad |\Delta y_{\text {j} \text {j}}| > 2.5 . \end{aligned}$$

The missing momentum is computed from the vectorial sum of the momenta of all the neutrinos present in the event. At fixed-order as well as at the LHE level each event contains exactly two charged leptons, however, when the QCD PS is included additional leptons can be generated by the decay of the hadrons: in the latter case, the cuts of Eq. (15) are applied to the two hardest leptons in the event. We only consider dressed leptons: photons are recombined with leptons if their relative distance in $\Delta R$ is smaller than 0.1.^3^ The jet candidates are reconstructed using the anti-$k_{\mathrm {T}}$ algorithm [64] with jet-resolution parameter $R=0.4$. The jet constituents are the coloured partons at fixed-order and LHE level, while for the results including PS and hadronisation effects jets are obtained from the final-state hadrons using the program Fastjet [65, 66]. Photons are recombined with jets if $\Delta R_{\text {j} \gamma }<0.1$. Along the line of Ref. [8], the tagging jets, which have to respect Eq. (17), are the two jets with highest transverse momentum that fulfil individually Eq. (16).

## Results and discussion

### Predictions for positive and negative same-sign W-boson scattering

**Cross sections**

We first report on the EW corrections for the processes $\text {p} \text {p} \rightarrow \mu ^+ \nu _\mu \text {e} ^+ \nu _{\text {e}} \text {j} \text {j}$ and $\text {p} \text {p} \rightarrow \mu ^- {\bar{\nu }}_\mu \text {e} ^- {\bar{\nu }}_{\text {e}} \text {j} \text {j}$. The cross sections at LO, NLO, and the relative corrections are listed in Table 1. While the cross sections deviate owing to the different partons in the initial state, the relative corrections are similar. The abundance of the $++$ signature with respect to the $--$ one is threefold at the LHC. On the other hand the relative corrections differ only by about one per cent. In Ref. [7], it has been shown that the large EW corrections to VBS are originating from large logarithms in the virtual corrections. Since these are related to the external states of the process, the relative correction factors in the logarithmic approximation are identical for both processes [7, 67]. Nonetheless, the typical scale of the process can deviate as the two processes possess different partonic channels with different associated PDFs. A variation in the scale (in the present case the invariant mass of the four leptons) implies thus (slightly) modified EW corrections. In particular, for the $++$ signature the average scale is $\langle M_{4\ell }\rangle \simeq 409 \,\text {GeV}$, while in the $--$ case it is $\langle M_{4\ell }\rangle \simeq 381 \,\text {GeV}$. Using the leading logarithmic approximation derived in Ref. [7], one obtains $-16.1\%$ and $-14.7\%$ for $++$ and $--$, respectively. This reproduces nicely the corrections for the full computations presented here. Note that the almost perfect agreement between the approximations and the full computations is somehow accidental given that the approximation is accurate only at the per-cent level.

With the code that we present the same-lepton-flavour cases can also be calculated. By computing the dominant partonic channels, we have found that the effect of interferences is marginal. For this reason, the results for the same lepton flavour are not shown in the present article.

**Table 1:** Cross sections at LO [$\mathcal {O}\left( \alpha ^6 \right)$] and NLO EW [$\mathcal {O}\left( \alpha ^7 \right)$] for $\text {p} \text {p} \rightarrow \mu ^+ \nu _\mu \text {e} ^+ \nu _{\text {e}} \text {j} \text {j}$ and $\text {p} \text {p} \rightarrow \mu ^- {\bar{\nu }}_\mu \text {e} ^- {\bar{\nu }}_{\text {e}} \text {j} \text {j}$ at the $13\,\text {TeV}$ LHC. The relative EW corrections are given in per cent, and the digits in parenthesis indicate the integration error

Process	$\sigma ^\mathrm{LO}$ [fb]	$\sigma ^\mathrm{NLO}_\mathrm{EW}$ [fb]	$\delta _\mathrm{EW}~[\%]$
$\text {p} \text {p} \rightarrow \mu ^+ \nu _\mu \text {e} ^+ \nu _{\text {e}} \text {j} \text {j}$	$\phantom {1}1.5345(1)$	$\phantom {1}1.292(2)$	$-15.8(1)$
$\text {p} \text {p} \rightarrow \mu ^- {\bar{\nu }}_\mu \text {e} ^- {\bar{\nu }}_{\text {e}} \text {j} \text {j}$	$\phantom {1}0.51832(3)$	$\phantom {1}0.4421(3)$	$-14.7(1)$

**Differential distributions**

**Fig. 2:**
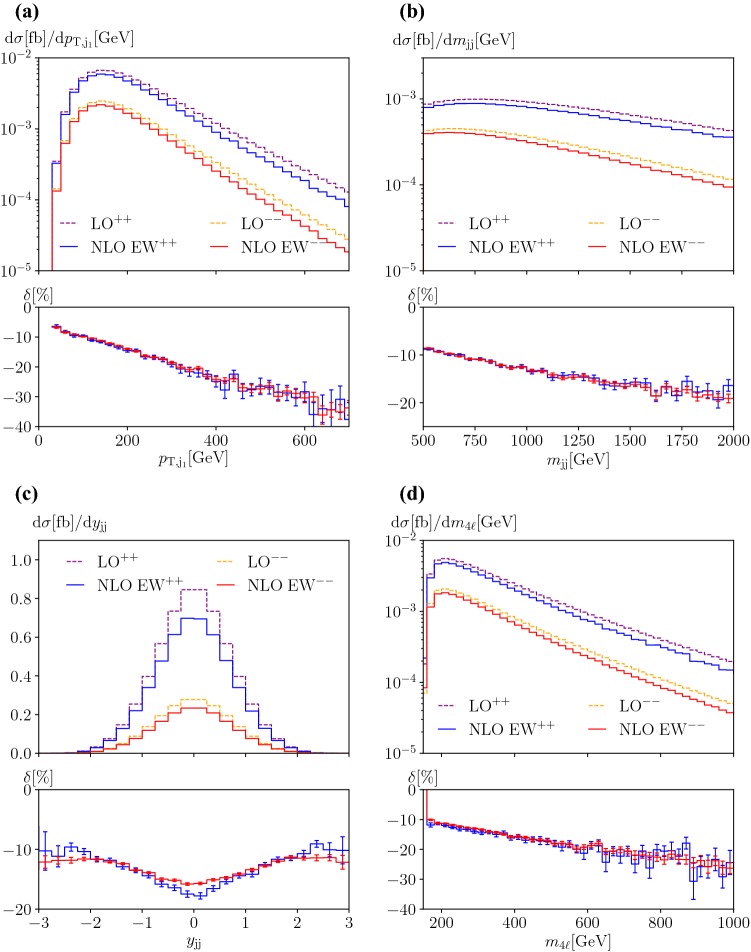
Differential distributions at LO [order $\mathcal {O}\left( \alpha ^6 \right)$] and NLO EW [order $\mathcal {O}\left( \alpha ^7 \right)$] for a centre-of-mass energy $\sqrt{s}=13\,\text {TeV}$ at the LHC for $\text {p} \text {p} \rightarrow \mu ^+\nu _\mu \text {e} ^+\nu _{\text {e}}\text {j} \text {j}$ and $\text {p} \text {p} \rightarrow \mu ^- {\bar{\nu }}_\mu \text {e} ^- {\bar{\nu }}_{\text {e}} \text {j} \text {j}$: **a** transverse momentum of the hardest jet (top left), **b** invariant mass of the two leading jets (top right), **c** rapidity of the two leading jets (bottom left), and **d** invariant mass of the four leptons (bottom right). The upper panels show the two LO contributions as well the two NLO predictions. The lower panels show the relative NLO corrections with respect to the corresponding LO in per cent

Some differential distributions for the processes $\text {p} \text {p} \rightarrow \mu ^+ \nu _\mu \text {e} ^+ \nu _{\text {e}} \text {j} \text {j}$ and $\text {p} \text {p} \rightarrow \mu ^- {\bar{\nu }}_\mu \text {e} ^- {\bar{\nu }}_{\text {e}} \text {j} \text {j}$ are presented in Fig. 2. For other distributions the corrections are qualitatively similar and differ only slightly in magnitude. In the upper plot, the absolute predictions are shown at LO and NLO EW for both signatures, while the lower plot displays the relative NLO EW corrections. The predictions for the $++$ signature are shown in dashed purple (LO) and solid blue (NLO), while the ones for the $--$ signature are drawn in dashed orange (LO) and solid red (NLO). The differential K-factors are coded in solid blue and red for the $+ +$ and $- -$ final state, respectively.

In Fig. 2a, the distribution in the transverse momentum of the hardest jet is shown. While the absolute predictions are clearly distinguishable for the two signatures, the relative corrections are practically identical. This is explained by the fact that the leading EW corrections factorise as shown in Ref. [7].

The invariant mass of the two tagging jets, which is displayed in Fig. 2b, is an observable that is often used as discriminant to define fiducial regions with enhanced EW contributions. As for the transverse momentum of the hardest jet, hardly any difference can be seen between the relative EW corrections for the two processes.

In Fig. 2c, the distribution in the rapidity of the two tagging jets is shown. This is the only observable that we have found where a visible difference emerges between the corrections of the two processes. In the central region, the corrections for the positive signature are negatively larger, while this is the opposite in the peripheral region. The differences are at the level of a couple of per cent.

Finally, we show the corrections for the distribution in the invariant mass of the four leptons. While this observable is not directly measurable experimentally, it is interesting from a theoretical point of view. In particular, this observable provides a good estimate for the typical scale of the VBS process. In addition, it is often used in new-physics analyses (see Refs. [68–70] for recent examples).

From these observations one could draw the conclusion that EW corrections for the two signatures of same-sign W-boson scattering are essentially the same. While this is the case for the considered particular set-up, it may not be true in general. Thus, if one wants to use the same corrections for the two processes, one should check that they are actually identical in the desired set-up. Finally, we have examined results for the same-lepton-flavour cases. We have not found any significant differences with respect to the different flavour cases. This shows that the effect of interference contributions is negligible.

### Comparison to previous computations

In this section we show comparative results between the newly implemented Powheg+Recola generator and MoCaNLO+Recola [43] which is one of the programs used for the original computation of Refs. [7, 8] of NLO EW corrections to $\text {p} \text {p} \rightarrow \mu ^+\nu _\mu \text {e} ^+\nu _{\text {e}}\text {j} \text {j}$. Besides comparing the cross section and differential distributions for the full partonic process at NLO EW accuracy, representative partonic channels have been checked individually. If not otherwise stated, the results for Powheg+Recola correspond to the stage 4 of the generation, *i.e.* after the emission of possibly multiple photons (the flag allrad 1 has been used). The results shown are obtained from about $600\,000$ events stored in LHE format. We note that despite being a rather large number of events, the corresponding statistical error is not particularly small. This is due to the fact that the events are generated completely inclusively, while the results shown here are only for a rather exclusive phase space.

**Fig. 3:**
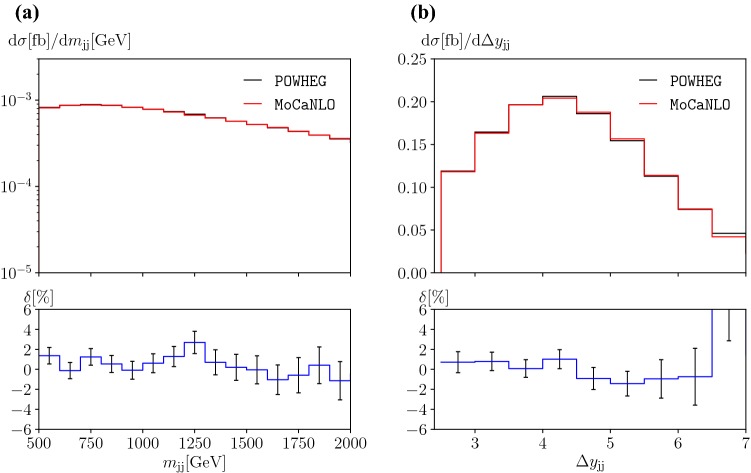
Comparison of differential distributions between Powheg+Recola (this work) and MoCaNLO+Recola (Ref. [7]) at NLO EW [order $\mathcal {O}\left( \alpha ^7 \right)$] at a centre-of-mass energy $\sqrt{s}=13\,\text {TeV}$ at the LHC for $\text {p} \text {p} \rightarrow \mu ^+\nu _\mu \text {e} ^+\nu _{\text {e}}\text {j} \text {j}$: **a** invariant mass of the two leading jets (left), and **b** rapidity difference of the two leading jets (right). The upper panels show the two NLO predictions. The lower panels display the relative difference between the two computations with the corresponding statistical error bars dominated by the Powheg+Recola predictions

In Table 2, fiducial cross sections at NLO EW, *i.e.* order $\mathcal {O}\left( \alpha ^7 \right)$, for the event selection described in Eqs. (15)–(17) are shown. In addition to the case where multiple photon radiation is possible, we also display the cross section for allrad 0 which is not significantly different. In all cases, statistical agreement is achieved against the independent computation of Ref. [7]. For the Powheg+Recola computation, the statistical error is around $0.3\%$ of the NLO result, while it is $0.05\%$ for the MoCaNLO+Recola computation.

**Table 2:** Cross sections at NLO EW [order $\mathcal {O}\left( \alpha ^7 \right)$] for $\text {p} \text {p} \rightarrow \mu ^+ \nu _\mu \text {e} ^+ \nu _{\text {e}} \text {j} \text {j}$ at the $13\,\text {TeV}$ LHC. These have been obtained with Powheg+Recola (this work, abbreviated P+R) with the flag allrad off/on and MoCaNLO+Recola (Ref. [7]). The digits in parenthesis indicate the integration error

Prediction	P+R allrad 0	P+R allrad 1	MoCaNLO+Recola
$\sigma ^\mathrm{NLO}_\mathrm{EW}$ [fb]	1.300(5)	1.302(5)	1.2895(6)

In addition to the cross section, we also present the comparison for differential distributions. Figure 3 shows the distributions in the invariant mass (Fig. 3a) as well as in the rapidity difference of the two tagging jets (Fig. 3b). These two observables are typically used in experimental analyses to enhance EW components over their QCD counterparts. The level of agreement is around few per cent for all bins. This corresponds to the statistical error of the Powheg+Recola computation. Other distributions display a similar level of agreement.

### Predictions at NLO EW accuracy in association with parton shower

**Fig. 4:**
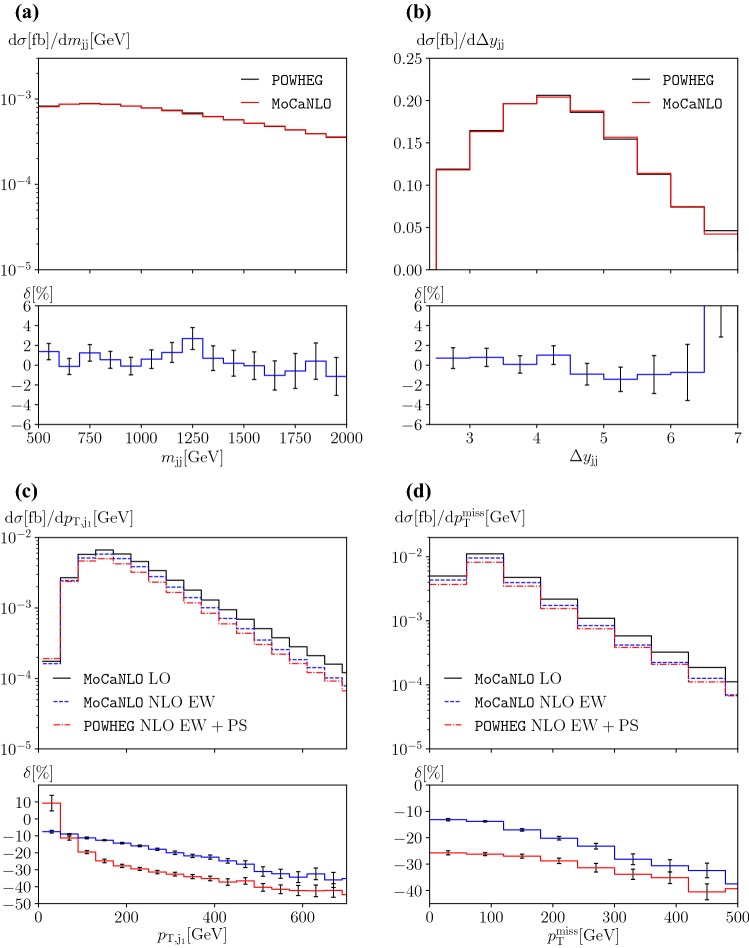
Differential distributions at LO [order $\mathcal {O}\left( \alpha ^6 \right)$], NLO EW [order $\mathcal {O}\left( \alpha ^7 \right)$] and NLO EW+PS at a centre-of-mass energy $\sqrt{s}=13\,\text {TeV}$ at the LHC for $\text {p} \text {p} \rightarrow \mu ^+\nu _\mu \text {e} ^+\nu _{\text {e}}\text {j} \text {j}$: **a** invariant mass of the two leading jets (top left), **b** rapidity difference of the two leading jets (top right), **c** transverse momentum of the hardest jet (bottom left), and **d** missing transverse energy (bottom right). The upper panels show the LO prediction as well as the NLO predictions with and without PS. The lower panels show the relative NLO corrections with respect to the corresponding LO in per cent

In this section, we show results at NLO EW and NLO EW+PS accuracy for illustrative purposes for the process $\text {p} \text {p} \rightarrow \mu ^+\nu _\mu \text {e} ^+\nu _{\text {e}}\text {j} \text {j}$. As explained in Sect. 3.1, the NLO EW corrections are matched to a QED PS and interfaced to a QCD PS. In particular, besides the PS evolution, hadronisation and decays of unstable hadrons are also taken into account. In Fig. 4, we restrict ourselves to a handful of distributions for brevity, but any distributions can be obtained from the code presented here. The phenomenological results concerning the PS effects are not new with respect to the in-depth study of Refs. [20, 43]. There, the effects of various PS and their matching to NLO QCD computations have been investigated in detail. The key improvement here is the combination of NLO EW corrections with PS and their availability in a public Monte Carlo program. We stress again that the present computation features the full matrix element at order $\mathcal {O}\left( \alpha ^6 \right)$, meaning that tri-boson and interference contributions are included throughout. In general the effects of PS are around ten per cent or more along the findings of Ref. [20]. Note that a one-to-one correspondence is not possible with the results of Ref. [20]. In the present computation the renormalisation and factorisation scales have been fixed to the W-boson mass and the shower scale to the geometric average of the jet transverse momenta. In Ref. [20], all scales have been set to the geometric average of the jet transverse momenta.

In the upper panels of Fig. 4, the predictions for the distributions at LO [order $\mathcal {O}\left( \alpha ^6 \right)$], NLO EW [order $\mathcal {O}\left( \alpha ^7 \right)$] and NLO EW+PS accuracy are shown. In the lower panel, the relative corrections normalised to the LO predictions together with their statistical errors are displayed for the NLO EW and NLO EW+PS predictions. The distributions in the invariant mass and in the rapidity difference of the two leading jets are depicted in Fig. 4a, b, respectively. The invariant-mass distribution features the typical Sudakov behaviour towards high energy which shows up as negatively increasing corrections. The effect of extra radiations translates into a lower acceptance rate towards high invariant masses. On the other hand, the distribution in the difference in the rapidity of the two tagging jets inherits mostly the overall NLO EW normalisation and decreases towards larger rapidity difference due to PS effects. Figure 4c, d display the distributions in the transverse momentum of the hardest jet and the missing momentum, respectively. Both show rather large EW corrections, reaching about $-30\%$ to $-40\%$ around $500\,\text {GeV}$ and beyond. The effect of extra radiation simulated by the PS tends to further lower the rate at high transverse momentum in both cases. The transverse momentum distribution of the hardest jet receives a large positive correction from the PS in the first bin. This is in agreement with the corresponding effect of the NLO QCD corrections [8]. It it due to the suppressed LO contribution in this bin and the reduction of the jet energy by radiation of gluons and photons. In general, the inclusion of the PS leads to a redistribution of events in phase space and pushes some fraction of events out of the fiducial phase space.

### Combination of EW corrections

In this article, we have presented a new generator able to compute EW corrections to VBS and to generate unweighted events. These predictions can be supplemented by photon and QCD radiation in parton/photon showers. The question arises, how these results can be combined with NLO QCD predictions.

Reference [71] provides prescriptions for the combination of NLO QCD and EW corrections matched to PS. We propose a modified version of the additive prescription of Ref. [71] that reads

18 $$ \begin{aligned} \left[ \frac{\mathrm{d} \sigma }{\mathrm{d} \mathcal {O}} \right] _{\mathrm{EW} \& \mathrm{QCD}}= & {} \left[ \frac{\mathrm{d} \sigma }{\mathrm{d} \mathcal {O}} \right] _{\mathrm{EW}+\mathrm{PS}}+ \left[ \frac{\mathrm{d} \sigma }{\mathrm{d} \mathcal {O}} \right] _{\mathrm{QCD}+\mathrm{QCD\, PS}}\nonumber \\&- \left[ \frac{\mathrm{d} \sigma }{\mathrm{d} \mathcal {O}} \right] _{\mathrm{LO}+\mathrm{QCD\, PS}}, \end{aligned}$$

where $\frac{\mathrm{d} \sigma }{\mathrm{d} \mathcal {O}}$ stands for the differential cross section as a function of the observable $\mathcal {O}$. The first term in Eq. (18) is what has been presented here, *i.e.* the NLO EW corrections matched to QED PS and supplemented with QCD PS with the strategy described in Sect. 3.3. The second term represents the predictions at NLO QCD matched to QCD PS only, as the inclusion of the QED PS would lead to a double counting of the mixed $\alpha \alpha _\mathrm{s}$ corrections already present in the first term of Eq. (18) in leading-logarithmic approximation. In order not to double count the LO matched to PS in the generators, it has to be subtracted (third term). From the above formulation, it is clear that the PS used in the three generators and that all input parameters should be identical in order to obtain consistent predictions.

Note that it is also possible to devise a multiplicative combination as in Ref. [71]. Nonetheless, we refrain from reproducing it here. Studying the effect of different combinations is beyond the scope of the present work and is thus left to upcoming work.

The predictions at NLO QCD matched to QCD PS can be obtained from public tools like MadGraph5_aMC@NLO [72], the vbf_wp_wp package of POWHEG-BOX-V2, or using VBFNLO as a matrix-element provider interfaced to a Monte Carlo event generator, as done in Ref. [73] for VBS $\text {W} ^+\text {W} ^-$ production. Note that the matrix elements from VBFNLO (that are also used in the vbf_wp_wp package of Powheg) have been obtained in the so-called VBS approximation [9, 10, 12]. While for current experimental precision such a level of accuracy is sufficient [20], for precision measurements the use of full computations as in Ref. [8] will be desirable.

## Conclusion

In this article we have presented a Monte Carlo event generator that allows to compute NLO EW corrections to same-sign W-boson scattering at the LHC and to generate unweighted events featuring these corrections. It is based on the Powheg Box framework in combination with Recola. Moreover, an interface to PYTHIA is provided. All relevant leptonic channels for the processes $\text {p} \text {p} \rightarrow \ell ^\pm _1 \nu _{\ell _1} \ell ^\pm _2 \nu _{\ell _2}\text {j} \text {j}$ are available and can be run easily.

We have exemplified the capabilities of the code: computing NLO EW corrections, generating unweighted events, and matching to parton/photon shower. Following Ref. [71], we have given a prescription to combine the present tool with existing tools for NLO QCD corrections matched to parton shower. This allows to reach NLO QCD+EW+PS accuracy which is the theoretical accuracy required for the VBS programme of the LHC for the next few years [6].

On the phenomenological side, we have computed for the first time the NLO EW corrections to all possible same-sign W-boson scattering processes, which were so far only known for the case of same-sign opposite-flavour leptons in the final state. While the total rates of the various channels are rather different, the corrections themselves are essentially identical in most relevant phase-space regions. This implies, in particular, that the interference effects between same- and different-lepton-flavour channels are rather suppressed.

On the technical side, we have demonstrated that Powheg+Recola works for a challenging $2 \rightarrow 6$ process featuring a non-trivial resonance structure. Recola is able to provide matrix elements for arbitrary processes at one loop in the SM and beyond. Thus, its combination with Powheg allows for the computation of NLO corrections matched to parton/shower for a large range of processes. The implementation that we have is rather simple and could be extended to more complex situations.

Finally, given the expected experimental accuracy for upcoming measurements, the use of such theoretical predictions/tools is becoming indispensable. We hope that experimental collaborations will make intensive use of them in order to exhaust the potential of the data taken at the LHC.

## Data Availability

This manuscript has associated data in a data repository. [Authors’ comment: It can be found at http://powhegbox.mib.infn.it/ under the directory User-Processes-RES/vbs-ssww-nloew.]

## Footnotes

1 A similar computation in a different framework has been performed in Ref. [28].

2 This particular PDF set does not have an identifier lhaid in the program LHAPDF6 [58].

3 In our predictions at NLO or at the LHE level with the flag allrad 0 this recombination prescription is equivalent to the one based on the anti-$k_{\mathrm {T}}$ algorithm used in Refs. [7, 8].
